# High-Energy Storage Performance in La-Doped Lead Zirconate Films on Flexible Mica Substrates

**DOI:** 10.3390/ma18102353

**Published:** 2025-05-19

**Authors:** Jianzeng Guo, Chao Yin, Xue Zhang, Qingguo Chi

**Affiliations:** 1 School of Electrical and Electronic Engineering, Harbin University of Science and Technology, Harbin 150080, China; guojianzeng0224@163.com (J.G.); zzx990617@163.com (X.Z.); qgchi@hotmail.com (Q.C.); 2 Key Laboratory of Engineering Dielectrics and Its Application, Ministry of Education, Harbin University of Science and Technology, Harbin 150080, China

**Keywords:** flexible, energy storage, (PbLa)ZrO_3_, ion doping, phase transition

## Abstract

Flexible thin-film capacitors have gained a lot of attention in energy storage applications because of their high energy storage densities and efficient charge–discharge performances. Among these materials, antiferroelectric compounds with low residual polarization and strong saturation polarization have shown great promise. However, their comparatively low breakdown strength continues to be a major issue restricting further developments in their energy storage performance. While La^3+^ doping has been explored as a means to enhance the energy storage capabilities of antiferroelectric thin films, the specific influence of La^3+^ on breakdown strength and the underlying mechanism of phase transitions have not been thoroughly investigated in existing research. In this study, Pb_1−3x/2_La_x_ZrO_3_ thin films were successfully synthesized and deposited on mica substrates via the sol–gel process. By varying the concentration of La^3+^ ions, a detailed examination of the films’ microstructures, electrical properties, and energy storage performances was carried out to better understand how La^3+^ doping influences both breakdown strength and energy storage characteristics. The results show that doping with La^3+^ significantly improves the breakdown strength of the films, reduces the critical phase transition electric field (*E*_F_-*E*_A_), and enhances their energy storage capabilities. Notably, the Pb_0.91_La_0.06_ZrO_3_ thin film achieved an impressive energy storage density of 34.9 J/cm^3^ with an efficiency of 58.3%, and at the maximum electric field strength of 1541 kV/cm, the recoverable energy density (*W*_rec_) was 385% greater than that of the PbZrO_3_ film. Additionally, the film still maintains good energy storage performance after 10^7^ cycles and 10^4^ bending cycles. These findings highlight the potential of flexible antiferroelectric Pb_0.91_La_0.06_ZrO_3_ thin films for future energy storage applications.

## 1. Introduction

As global energy demand keeps rising and climate change brings more and more challenges, the development of effective and environmentally friendly energy storage systems has turned into a crucial technological approach to dealing with energy shortages and lessening the impacts of climate change. In the area of energy storage, as a prospective means of electrical energy storage, inorganic dielectric capacitors have attracted much attention. Because of their extraordinary efficiency in charge–discharge processes, and high energy density, dielectric capacitors are known for their high efficiency in charge–discharge processes as well as their outstanding stability [1,2,3,4,5]. Despite their excellent charge–discharge rates and cycling stability, the application of dielectric capacitors in large-scale engineering projects is still restricted by their relatively low energy storage capacity [6,7,8,9,10,11]. Consequently, increasing the energy storage density of dielectric capacitors has turned into a major focus of research [12,13,14]. Energy storage density is an important parameter for assessing the energy storage performance of dielectric capacitors. It represents the quantity of energy stored per unit volume of the dielectric material under an applied electric field [15,16]. In the case of nonlinear dielectrics, the equations provided below can be used to calculate the efficiency (*η*) using the overall energy storage density (*W*_tot_) and the density of recoverable energy (*W*_rec_) [17].

(1) $$W_{\mathrm{tot}}=\int_{0}^{P_{m}}EdP$$

(2) $$W_{\mathrm{rec}}=\int_{P_{r}}^{P_{m}}EdP$$

(3) $$\eta =\frac{W_{\mathrm{rec}}}{W_{\mathrm{tot}}}=\frac{W_{\mathrm{rec}}}{W_{\mathrm{rec}}+W_{\mathrm{loss}}}$$

*P*_m_ and *P*_r_ represent the saturation polarization and remnant polarization, respectively; *W*_loss_ is the energy loss during the charge–discharge process, and *E* is the applied electric field [18].

Lead zirconate (PbZrO_3_), a well-established antiferroelectric material, is commonly used in dielectric capacitors due to its near-zero remnant polarization and high saturation polarization when subjected to an external electric field [19,20]. However, its relatively low breakdown strength remains a significant challenge to improving its energy storage performance. In recent years, element doping has attracted significant interest as a promising approach to improving the energy storage performance and stability of thin films [21,22]. For instance, Ye et al. synthesized Eu-doped PbZrO_3_ thin films using the sol–gel technique and demonstrated that the incorporation of Eu influenced both the Curie temperature and the phase transition electric field [23]. At a doping concentration of 3%, the maximum energy storage density achieved was 18.8 J/cm^3^. Sa et al. investigated the antiferroelectric–ferroelectric phase transition process by incorporating tungsten (W) elements into PbZrO_3_ [24]. As the W content increased, lattice distortion occurred, the film orientation shifted from (111) to (110), and the Curie temperature progressively decreased. However, while ion doping has improved the performance of thin films, enhancing their flexibility and sustainability remains a critical challenge. Mica, a layered silicate mineral with exceptional mechanical flexibility and thermal stability, has become an ideal substrate for flexible films due to its superior physical properties [17,25]. Flexible inorganic energy storage thin films fabricated on Mica substrates not only retain the flexibility of Mica but also integrate the excellent energy storage performance of PbZrO_3_, enabling efficient energy storage [26]. In this study, Pb_1−3x/2_La_x_ZrO_3_ antiferroelectric thin films were fabricated on Mica substrates using the sol–gel method, aiming to improve the films’ energy storage density and achieve flexibility. By adjusting the La^3+^ doping concentration (x = 0, 0.04, 0.06, 0.08), the effects of La^3+^ doping on the microstructure and antiferroelectric properties of the PbZrO_3_ thin films were thoroughly investigated. The mechanisms through which La^3+^ doping affects the energy storage performance of the films were analyzed, and the changes in energy storage behavior under external electric fields were investigated.

The results demonstrate that the breakdown strength of the Pb_0.91_La_0.06_ZrO_3_ thin film reached a maximum value of 1541.0 kV/cm, which is 3.1 times higher than that of the PbZrO_3_ thin film (492.5 kV/cm). Additionally, the film exhibited optimal energy storage performance, with a *W*_rec_ of 34.9 J/cm^3^ and an *η* of 58.3%. The objective of this research is to investigate the effect of La^3+^ doping on the breakdown strength, dielectric properties, and energy storage performance of PbZrO_3_ thin films. While previous studies have explored the impact of doping on the energy storage performance of antiferroelectric materials, the specific mechanisms behind the enhancement of breakdown strength and phase transitions remain inadequately understood. This study aims to fill this gap by systematically varying the La^3+^ doping concentration and examining its influence on the material properties of the films, thereby providing a deeper understanding of the role of La^3+^ doping in enhancing the performance of PbZrO_3_ thin films.

## 2. Experimental Section

### 2.1. Film Preparation

The Pb_1−3x/2_La_x_ZrO_3_ precursor solution was synthesized by dissolving stoichiometric quantities of Pb(CH_3_COO)_2_·5H_2_O (99.99%, Beijing InnoChem Science & Technology Co., Ltd., Beijing, China) and La(NO_3_)_3_·6H_2_O (99%, Beijing InnoChem Science & Technology Co., Ltd.) in a binary solvent system comprising CH_3_OCH_2_CH_2_OH (99%, Beijing InnoChem Science & Technology Co., Ltd.) and CH_3_COOH (99.5%, Beijing InnoChem Science & Technology Co., Ltd.), with zirconium isopropoxide (C_12_H_28_O_4_Zr (70%, Beijing InnoChem Science & Technology Co., Ltd.)) introduced as the liquid-phase precursor. To compensate for lead loss during high-temperature annealing, an additional 10% of lead was incorporated. The mixture was stirred at ambient temperature for one hour, followed by storage at a cool temperature for 24 h, resulting in a clear and transparent Pb_1−3x/2_La_x_ZrO_3_ precursor solution [27,28,29]. For substrate preparation, the Mica was meticulously peeled using tweezers to achieve a smooth surface. It was then ultrasonically cleaned in anhydrous ethanol to eliminate surface impurities. After drying, a Pt electrode was deposited on the substrate via magnetron sputtering. The precursor solution was applied to the Mica substrate through spin coating, initially at 1000 rpm for 10 s, followed by 3000 rpm for 20 s to form a wet film. This film was then dried on a hot plate at 400 °C for 3 min to ensure complete removal of the organic solvents. The spin-coating process was repeated until the desired thickness of the inorganic thin film was achieved. Finally, the dried film was transferred to a rapid thermal annealing furnace for crystallization. The furnace’s temperature program was configured to rapidly reach the target annealing temperature, which was maintained for 3 min to ensure complete crystallization of the thin film. Figure S1 shows a flowchart of the thin film preparation method. The preparation of precursors and inorganic energy storage films is described in detail.

### 2.2. Characterization

The phase structures of the thin films and ceramic powders were analyzed using a PANalytical XRD-600 X-ray diffractometer (Almelo, The Netherlands). Raman scattering spectra for the thin-film samples were obtained with a Renishaw inVia Raman spectrometer (Renishaw, Gloucestershire, UK), covering a frequency range from 100 cm^−1^ to 800 cm^−1^. The cross-sectional morphology and thickness of the thin films were examined with a field-emission scanning electron microscope (SEM, SU8020, Hitachi, Tokyo, Japan). The surface topography and roughness of the thin films were studied using an atomic force microscope (AFM, Dimension Icon, Bruker, Bremen, Germany). To evaluate the ferroelectric properties, the hysteresis loops and leakage current density of the thin-film samples were measured using a Radiant Premier II ferroelectric performance testing system (Radiant, Redmond, WA, USA).

## 3. Results and Discussion

**Figure 1:**
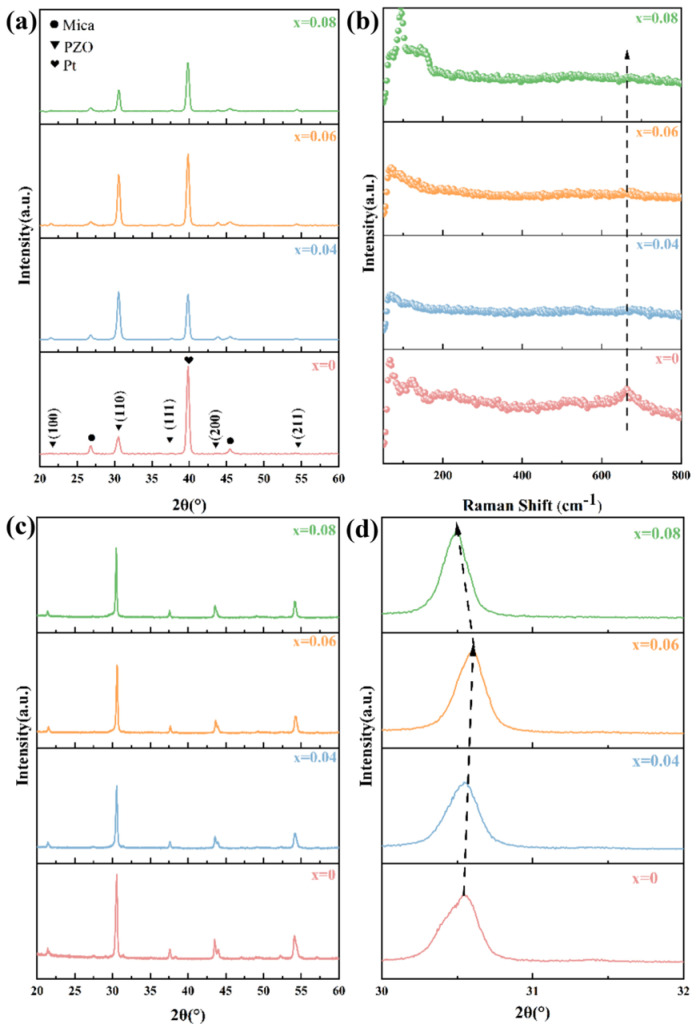
Microstructural characterization of Pb_1−3x/2_La_x_ZrO_3_ thin films. (**a**) XRD patterns. (**b**) Raman spectroscopy. (**c**,**d**) XRD patterns of powder.

Figure 1a displays the XRD spectra of the Pb_1−3x/2_La_x_ZrO_3_ films (x = 0, 0.04, 0.06, 0.08). The results demonstrate that films with varying La^3+^ doping concentrations all exhibit a perovskite structure, preferentially oriented along the (110) direction. When the La^3+^ doping concentration reaches 0.08, the intensity of the (110) diffraction peak of the Pb_0.88_La_0.08_ZrO_3_ film decreases, possibly due to changes in La^3+^ occupancy as the doping content increases [30,31,32,33]. Figure 1b displays the Raman spectrum of the Pb_1−3x/2_La_x_ZrO_3_ films. The Raman peak at 664 cm^−1^ corresponds to the Zr-O bond stretching vibration mode, and the intensity of this characteristic peak gradually decreases as the La^3+^ doping concentration increases. Figure S2 presents the complete Raman spectra. In this study, Pb_1−3x/2_La_x_ZrO_3_ ceramic powders were prepared, and the XRD spectrum is shown in Figure 1c. No secondary peaks were observed in the XRD patterns, confirming that La^3+^ has been fully solid-solubilized. Figure 1d displays the detailed spectrum of the (110) diffraction peak, with a scanning range of 30–32°. As the La^3+^ doping concentration increases, the (110) diffraction peak shifts to higher angles. When the La^3+^ doping concentration reaches 0.08, the diffraction peak shifts to lower angles. According to the Bragg equation, *nλ* = 2*d*sinθ, the interplanar spacing (*d*) first increases and then decreases as the La^3+^ concentration increases. The ionic radius of La^3+^ is 0.103 nm, that of Pb^2+^ is 0.119 nm, and that of Zr^4+^ is 0.072 nm. At low doping concentrations, La^3+^ tends to occupy the Pb^2+^ sites, resulting in a reduction in the lattice constant. However, when the doping concentration reaches 0.08, the occupancy of La^3+^ may change, with La^3+^ preferentially occupying the Zr^4+^ sites, resulting in an increase in the interplanar spacing. Figure S3 shows a transmission electron microscope image of Pb_0.88_La_0.08_ZrO_3_ film. Through measurements, it was found that the interplanar spacing of Pb_0.88_La_0.08_ZrO_3_ is 0.35 nm, which is significantly larger than the interplanar spacing of PbZrO3, which is 0.31 nm [17].

**Figure 2:**
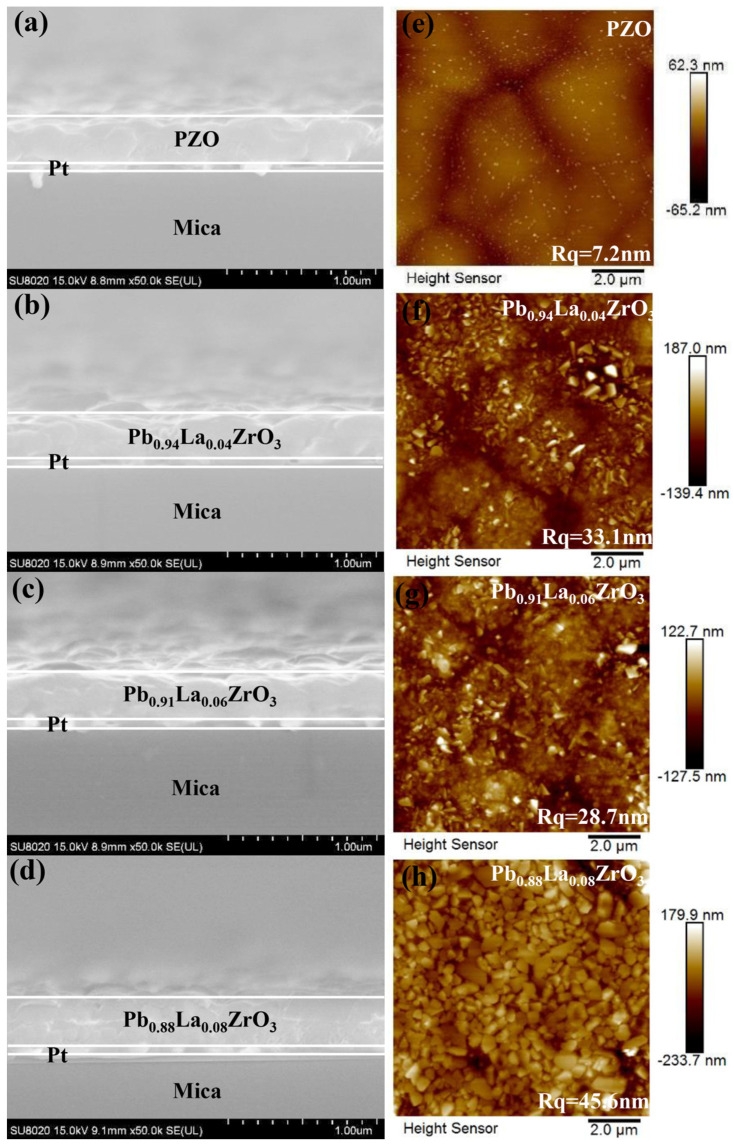
The cross-sectional SEM images and AFM images of Pb_1−3x/2_La_x_ZrO_3_ films. (**a**) SEM image of PbZrO_3_ film. (**b**) SEM image of Pb_0.94_Zr_0.04_O_3_ film. (**c**) SEM image of Pb_0.91_Zr_0.06_O_3_ film. (**d**) SEM image of Pb_0.88_Zr_0.08_O_3_ film. (**e**) AFM image of PbZrO_3_ film. (**f**) AFM image of Pb_0.94_Zr_0.04_O_3_ film. (**g**) AFM image of Pb_0.91_Zr_0.06_O_3_ film. (**h**) AFM image of Pb_0.88_Zr_0.08_O_3_ film.

Figure 2a–d display the cross-sectional SEM images of Pb_1−3x/2_La_x_ZrO_3_ films. The images indicate that the cross-sectional structure of the films is intact, with no significant defects such as pores or cracks, and the film thickness is approximately 260 nm. Figure 2e–h present the surface AFM images of Pb_1−3x/2_La_x_ZrO_3_ films. The particle size is uniform, and the film surface appears dense. As La^3+^ is introduced, the surface roughness of the films tends to increase [34,35]. The root mean square (RMS) surface roughness values of the Pb_1−3x/2_La_x_ZrO_3_ films (x = 0, 0.04, 0.06, 0.08) are 7.2 nm, 33.1 nm, 28.7 nm, and 45.6 nm, respectively [36,37]. Figure S4 shows the elemental mapping of the Pb_1−3x/2_La_x_ZrO_3_ film. The presence of various constituent elements in the Pb0.91La0.06ZrO3 thin film can be clearly observed in the elemental mapping images.

**Figure 3:**
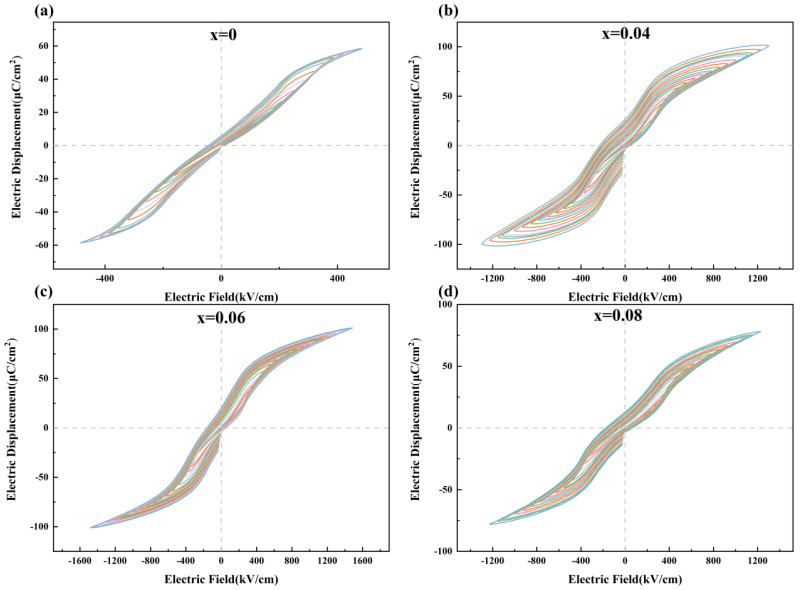
The *P*-*E* loops of Pb_1−3x/2_La_x_ZrO_3_ films. (**a**) x = 0 (**b**) x = 0.04 (**c**) x = 0.06 (**d**) x = 0.08.

Figure 3 presents the *P*-*E* loops of the Pb_1−3x/2_La_x_ZrO_3_ (x = 0, 0.04, 0.06, 0.08) films. The figure indicates that the films display characteristic double hysteresis loops. As the La^3+^ doping concentration increases, the breakdown strength of the films improves, and the *P*_m_ first increases and then decreases with higher doping concentrations [38,39].

**Figure 4:**
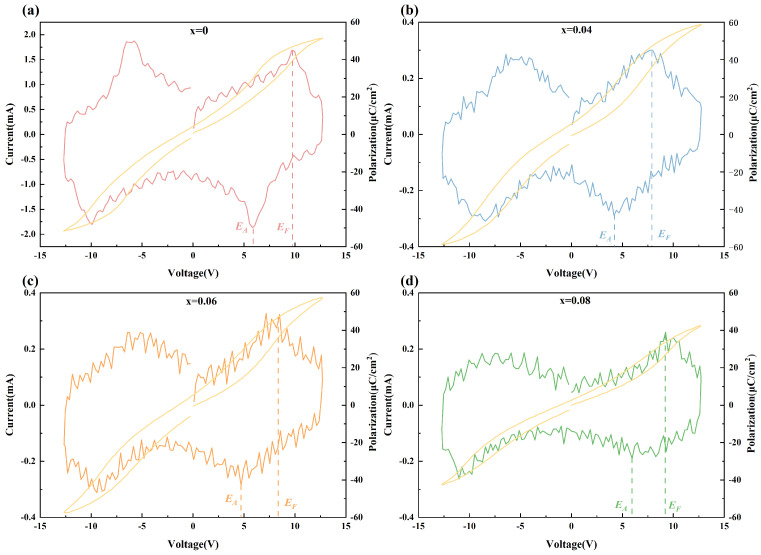
The *P-E* loops and polarization of Pb_1−3x/2_La_x_ZrO_3_ films. (**a**) x = 0 (**b**) x = 0.04 (**c**) x = 0.06 (**d**) x = 0.08.

Figure 4 presents the polarization current and hysteresis loops of the Pb_1−3x/2_La_x_ZrO_3_ films under an identical electric field. The Pb_1−3x/2_La_x_ZrO_3_ films display four current peaks, consistent with their antiferroelectric characteristics. *E*_F_ and *E*_A_ correspond to the critical electric fields for the antiferroelectric-to-ferroelectric and ferroelectric-to-antiferroelectric phase transitions of the films, respectively. Furthermore, the incorporation of La^3+^ results in a decrease in the polarization current [19].

**Figure 5:**
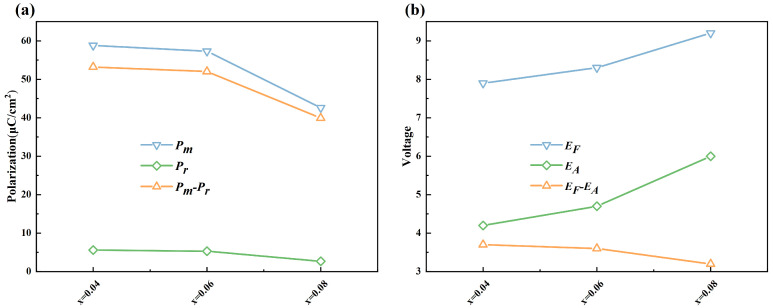
The polarization of Pb_1−3x/2_La_x_ZrO_3_ films. (**a**) Comparison of polarization of the films. (**b**) Phase transition electric fields of the films.

Figure 5 illustrates the variation in the polarization and critical phase transition electric fields of the films with varying La^3+^ doping concentrations under an identical electric field. The *P*_m_ and *P*_r_ exhibit a distinct decreasing trend with an increasing La^3+^ doping concentration [40,41,42,43]. Concurrently, the *P*ₘ–*P*ᵣ value also decreases, which is attributed to the ion vacancies generated during the doping process. As the La^3+^ doping concentration increases, the values of *E*_F_ and *E*_A_ gradually increase, which contributes to the enhancement of the film’s *W*_rec_. Furthermore, the *E*_F_–*E*_A_ value gradually decreases, which is consistent with the trend observed in the film’s *P*-*E* loops shown in Figure 3. The decrease in the *E*_F_–*E*_A_ value indicates that the energy loss during the field-induced phase transition process is relatively small, which is advantageous for improving the *η* of the films.

**Figure 6:**
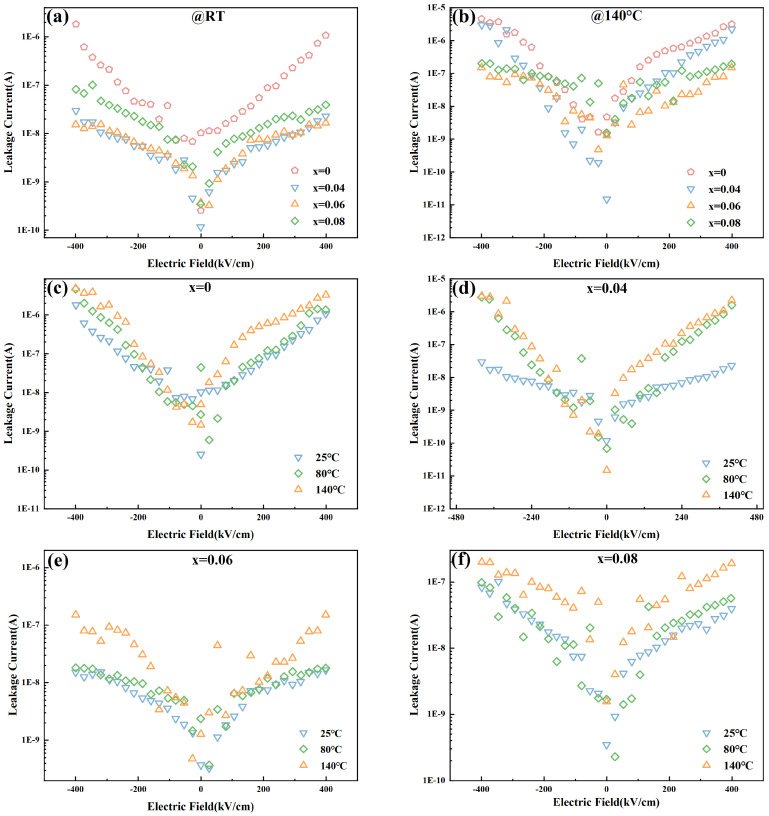
The leakage current of Pb_1−3x/2_La_x_ZrO_3_ films at different temperatures. (**a**) Leakage current at room temperature. (**b**) Leakage current at 140 °C. (**c**) Leakage current of PbZrO_3_ film at different temperatures. (**d**) Leakage current of Pb_0.94_Zr_0.04_O_3_ film at different temperatures. (**e**) Leakage current of Pb_0.91_Zr_0.06_O_3_ film at different temperatures. (**f**) Leakage current of Pb_0.88_Zr_0.08_O_3_ film at different temperatures.

Figure 6a,b present a comparison of the leakage current of the Pb_1−3x/2_La_x_ZrO_3_ films at room temperature and 140 °C. At room temperature, as the La^3+^ doping concentration increases, the leakage current first decreases and then increases [44]. On the one hand, La^3+^ doping captures mobile charge carriers, leading to a decrease in leakage current. However, when the doping concentration reaches 0.08, the leakage current increases. This is attributed to a significant increase in the number of oxygen vacancies at higher doping concentrations, which enlarge the conduction path for electrons, resulting in a decline in their insulating properties [45,46]. At 140 °C, the leakage current for the film with a La^3+^ concentration of x = 0.04 is relatively high, likely due to the insufficient effect of the lower La^3+^ concentration on conductivity. This allows charge carriers to migrate more freely at elevated temperatures, leading to higher conductivity and, consequently, an increased leakage current. Conversely, the leakage current for the film with a La^3+^ concentration of x = 0.06 is the lowest, which can be attributed to the doping concentration approaching the “saturation point”. At this concentration, La^3+^ ions induce significant lattice distortion, charge recombination, and local electric field effects, thereby minimizing the leakage current [47,48,49]. Figure 6c–f present a comparison of leakage current in Pb_1−3x/2_La_x_ZrO_3_ films at different temperatures. As the temperature increases, the leakage current exhibits an upward trend, but the change in leakage current with temperature is minimal, demonstrating the excellent temperature stability of Pb_1−3x/2_La_x_ZrO_3_ films [50].

**Figure 7:**
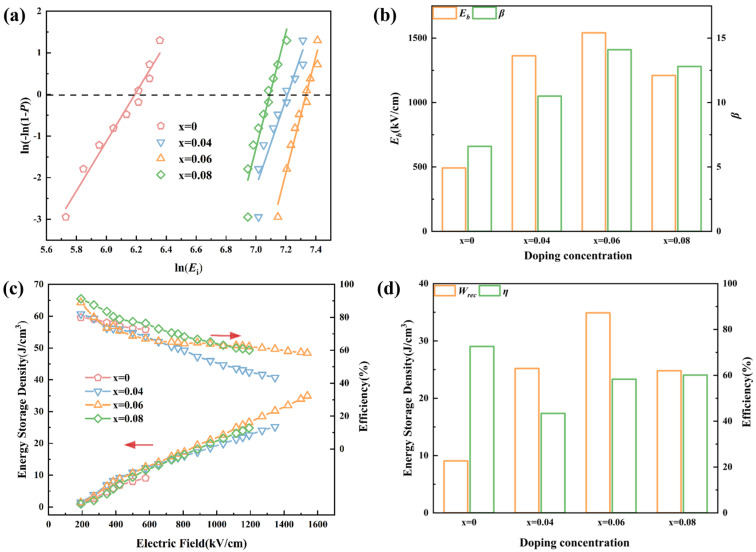
Electrical and energy storage performance of Pb_1−3x/2_La_x_ZrO_3_ films. (**a**,**b**) Weibull distribution of the *E*_b_ of Pb_1−3x/2_La_x_ZrO_3_ films. (**c**,**d**) Energy storage properties of Pb_1−3x/2_La_x_ZrO_3_ thin films.

Figure 7a,b present the Weibull distribution of the *E*_b_ of Pb_1−3x/2_La_x_ZrO_3_ films. The results indicate that following La^3+^ doping, the *E*_b_ of the films increases significantly. Specifically, the breakdown strengths of the Pb_1−3x/2_La_x_ZrO_3_ (x = 0, 0.04, 0.06, 0.08) films are 492.5 kV/cm, 1363.0 kV/cm, 1541.0 kV/cm, and 1210 kV/cm, respectively, with shape parameters (*β*) of 6.6, 10.5, 14.1, and 12.8. The Pb_0.91_La_0.06_ZrO_3_ film exhibits the highest *E*_b_ and *β*. The trend in breakdown strength with increasing La^3+^ doping is consistent with the trend observed for leakage current [51,52]. A lower doping concentration benefits the insulating properties of the films, while excessive doping leads to a gradual deterioration in their breakdown strength. Figure 7c,d present the *W*_rec_ of Pb_1−3x/2_La_x_ZrO_3_ films at the maximum electric field strength. The *W*_rec_ values are 9.07 J/cm^3^, 25.2 J/cm^3^, 34.9 J/cm^3^, and 24.8 J/cm^3^, respectively. Among these, the *W*_rec_ of Pb_0.91_La_0.06_ZrO_3_ films is 385% higher than that of PbZrO_3_. At the maximum electric field strength, the *η* of Pb_1−3x/2_La_x_ZrO_3_ films are 72.6%, 43.4%, 58.3%, and 60.1%, respectively. The improvement in energy storage performance can be attributed to two primary factors [53,54]. On the one hand, La^3+^ doping enhances the breakdown strength of the films, thereby increasing their *W*_rec_. On the other hand, the decrease in the critical phase transition electric field (*E*_F_-*E*_A_) of the films leads to an increase in *η*. Figure S5 shows results of the bending and electrocyclic stability test for the Pb_0.91_La_0.06_ZrO_3_ film. After 10^4^ bending cycles, the fluctuations in *W*_rec_ and *η* are minimal. After 10^7^ cycles, the energy storage performance of the film capacitor shows almost no change, indicating that the film capacitor exhibits excellent cycling and bending stability.

## 4. Conclusions

In summary, Pb_1−3x/2_La_x_ZrO_3_ films were successfully fabricated on Mica substrates using the sol–gel method, and their microstructures, electrical properties, and energy storage performances were systematically examined. Pb_0.91_La_0.06_ZrO_3_ films achieved a remarkable *W*_rec_ of 34.9 J/cm^3^ and an *η* of 58.3%, and at the maximum electric field strength of 1541 kV/cm, the *W*_rec_ is 385% higher than that of the PbZrO_3_ film. The remarkable energy storage performance of Pb_0.91_La_0.06_ZrO_3_ films can be attributed to the improved breakdown strength and the reduced *E*_F_-*E*_A_. The specific mechanism for the enhanced breakdown strength and phase transition is explained as follows. On the one hand, the La^3+^ doping process enhances the breakdown strength of the films, thereby increasing their *W*_rec_. On the other hand, the reduction in the *E*_F_-*E*_A_ of the films leads to an increase in *η*. Additionally, the film still maintains a good energy storage performance after 10^7^ electric cycles and 10^4^ bending cycles. These exceptional energy storage properties highlight the significant potential of flexible Pb_1−3x/2_La_x_ZrO_3_ thin-film capacitors in future energy storage device applications.

## Data Availability

The data that support the findings of this study are available from the corresponding authors upon reasonable request. The data are not publicly available due to data privacy principles.

## Supplementary Materials

The following supporting information can be downloaded at: https://www.mdpi.com/article/10.3390/ma18102353/s1, A flow chart of the thin film preparation, complete with Raman spectra, stability tests and transmission electron microscopy and elemental mapping annealing time curve and energy storage comparison table is available in the Supporting Information. Figure S1: Flow chart of precursor preparation and film preparation; Figure S2: Complete Raman spectra of Pb_0.91_La_0.06_ZrO_3_ thin films; Figure S3: Transmission electron micrographs of Pb_0.88_La_0.08_ZrO_3_ films; Figure S4: Elemental mapping images of Pb_0.91_La_0.06_ZrO_3_ thin films; Figure S5: Stability of Pb_0.91_La_0.06_ZrO_3_ film at 800 kV/cm; Figure S6: Annealing temperature and time curve; Table S1: A comparison of the energy storage performance of this work with other energy storage materials; References [6,55,56,57,58,59,60,61] are cited in the Supplementary Materials.
