# Supplementary material for: High-Energy Storage Performance in La-Doped Lead Zirconate Films on Flexible Mica Substrates
Source: Materials (Basel). 2025 May 19;18(10):2353. doi: 10.3390/ma18102353 (PMC12112967; doi:10.3390/ma18102353)
Supplement: Supplementary file 1 [file materials-18-02353-s001.zip › materials-3469845-supplementary.pdf]

Supporting Information

High-Energy Storage Performance in La-Doped Lead Zirconate Films on Flexible Mica Substrates

Jianzeng Guo [1,2], Chao Yin [1,2,*], Xue Zhang [1,2] and Qingguo Chi [1,2]

[1] School of Electrical and Electronic Engineering, Harbin University of Science and Technology,
Harbin 150080, China; guojianzeng0224@163.com (J.G.); zzx990617@163.com (X.Z.);
qgchi@hotmail.com (Q.C.)

[2] Key Laboratory of Engineering Dielectrics and Its Application, Ministry of Education, Harbin University of Science and Technology, Harbin 150080, China

* Correspondence: cyin@hrbust.edu.cn

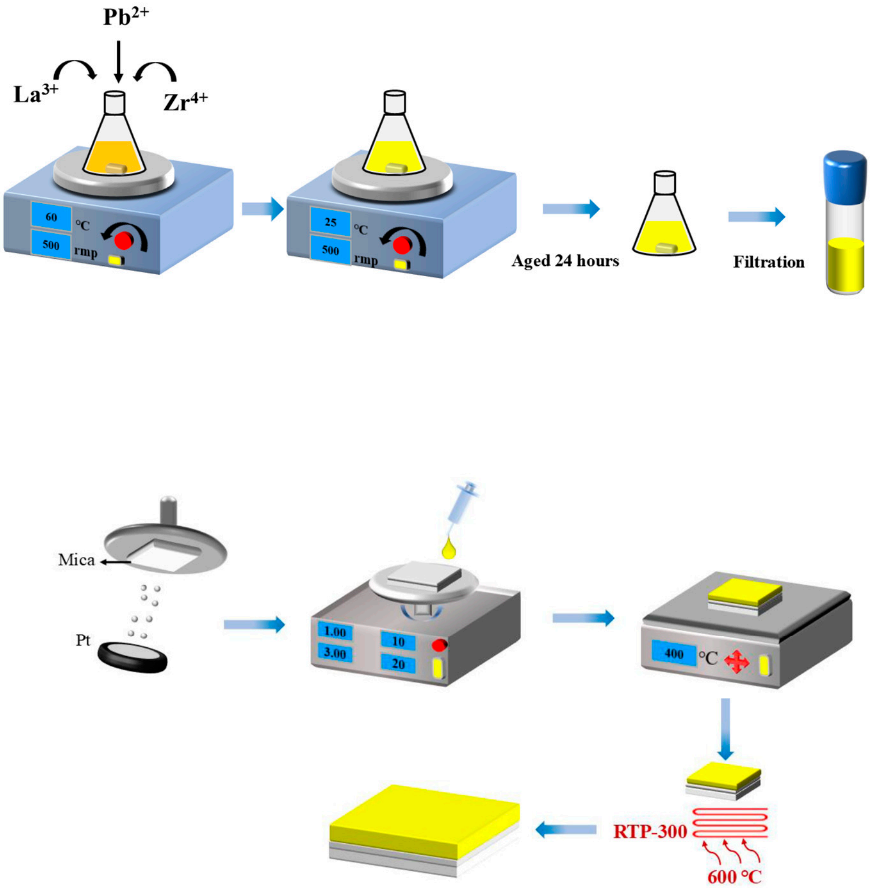

Figure S1. Flow chart of precursor preparation and film preparation.

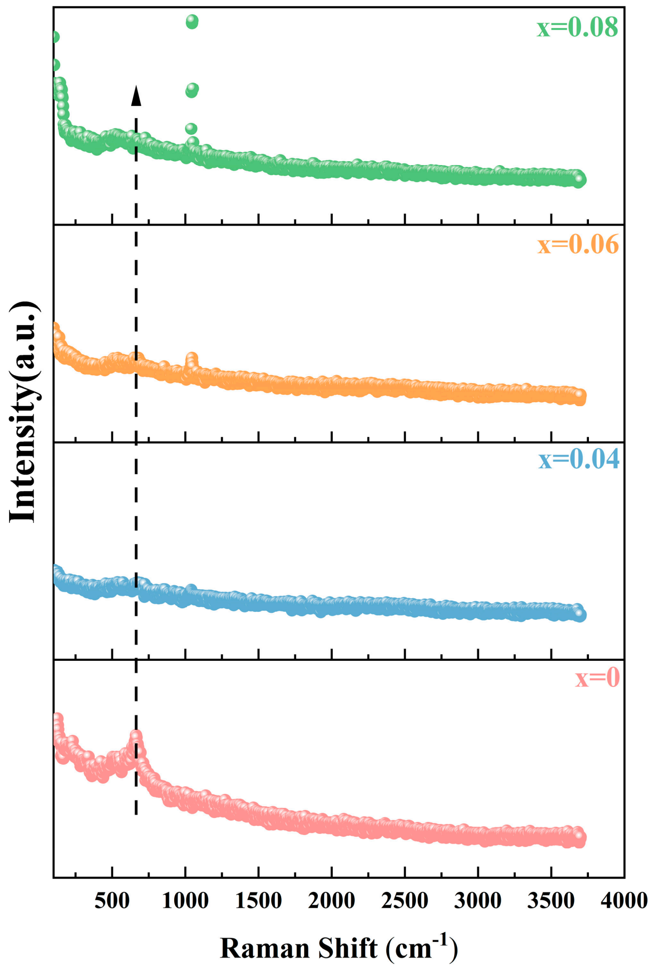

Figure S2. Complete Raman spectra of $Pb_{0.91}La_{0.06}ZrO_3$ thin films.

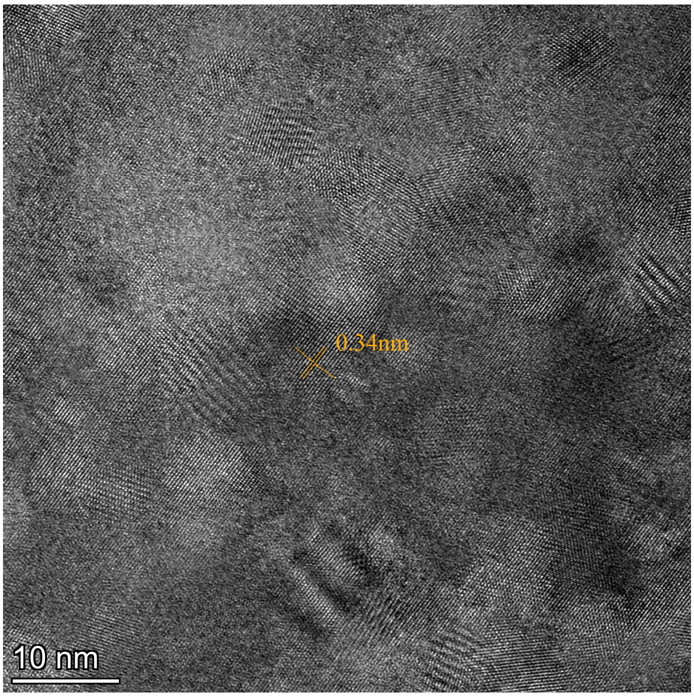

Figure S3. Transmission electron micrographs of $Pb_{0.88}La_{0.08}ZrO_3$ films.

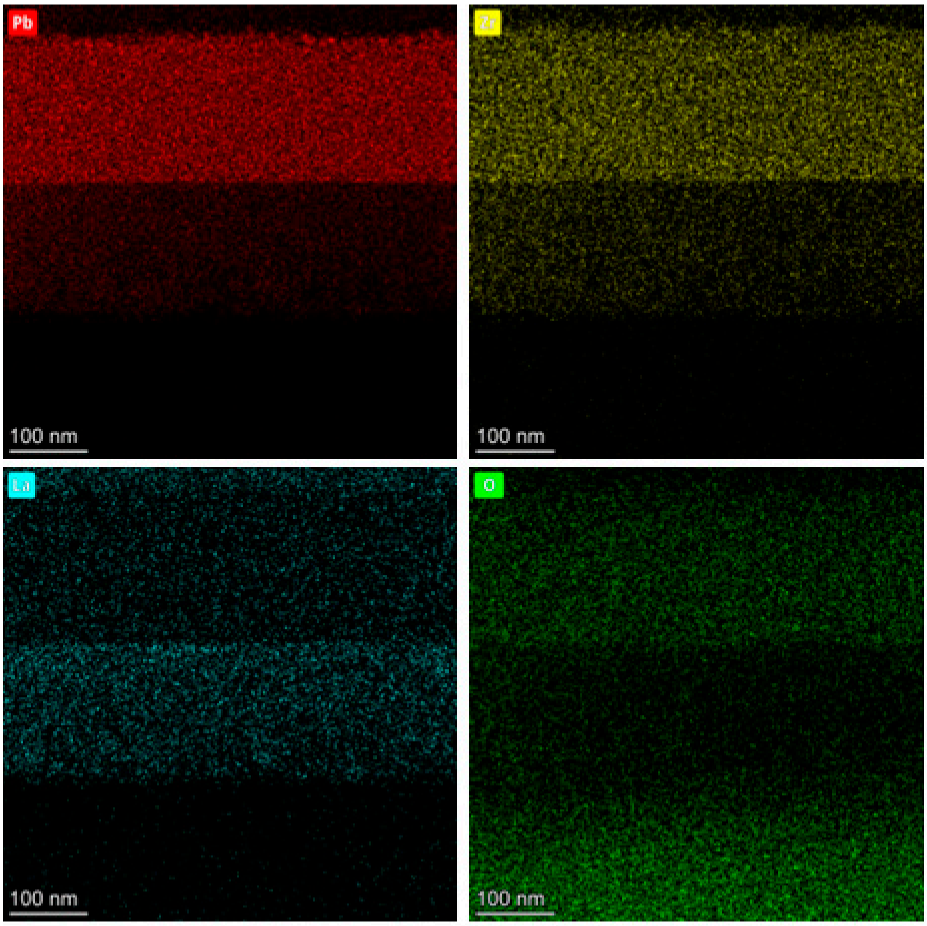

Figure S4. Elemental mapping images of $Pb_{0.91}La_{0.06}ZrO_3$ thin films.

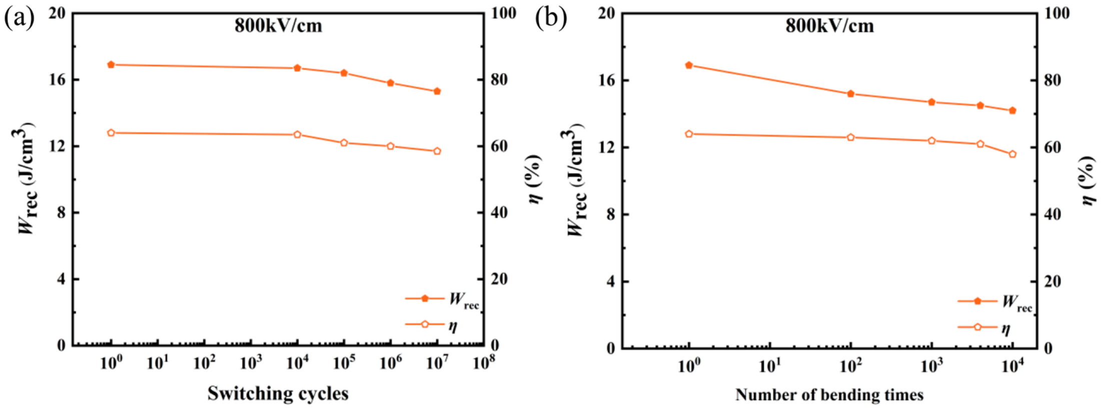

Figure S5. Stability of $Pb_{0.91}La_{0.06}ZrO_3$ film at 800 kV/cm (a) Electrical cycling stability. (b) Bending stability.

Figure S5. shows the bending and electrocyclic stability test of the $Pb_{0.91}La_{0.06}ZrO_3$ film. It can be seen that the energy storage performance of the film changes little after the cycle of 10^7 and the bending of 10^4, so the film has good bending stability and cycle stability.

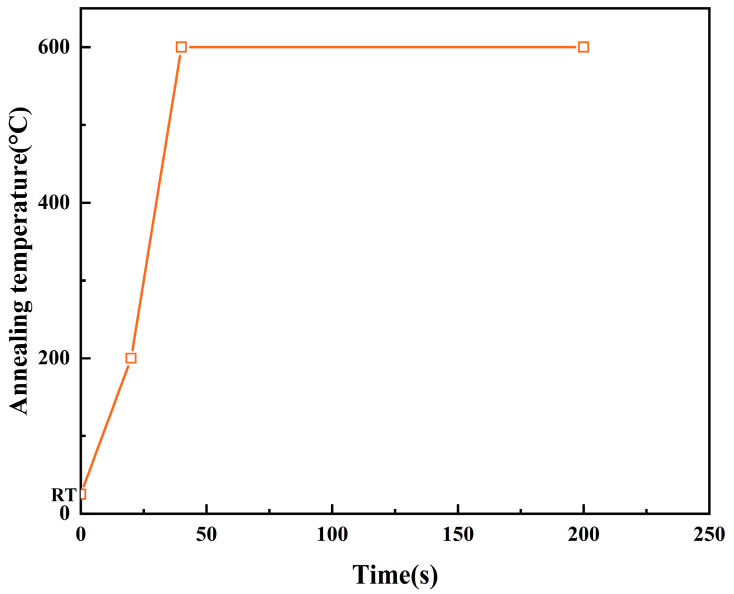

Figure S6. Annealing temperature and time curve.

Materials	W_{rec}（J/cm^3）	η（%）	Reference
$Pb_{0.91}La_{0.06}ZrO_3$	34.9	58.3	this work
PZO	16.6	50.4	[6]
PBLZST	6.8	61.2	[55]
PZO-PSO	6.1	72.1	[56]
PLZT	11.4	50.0	[57]
PLZST	2.0	71	[58]
PLZS	4.8	82.5	[59]
PZ-PMM	0.6	--	[60]
PLZST	1.3	78.7	[61]

Table S1. A comparison of the energy storage performance of this work with other energy storage materials.

[6] Yin C, Zhang T D, Zhang B W, et al. High energy storage performance for flexible $PbZrO_3$ thin films by seed layer engineering [J]. Ceramics International, 2022, 48(16): 23840-23848.

[55] Chen L M, Li Y, Zhang Q W, et al. Electrical properties and energy-storage performance of $(Pb_{0.92}Ba_{0.05}La_{0.02})(Zr_{0.68}Sn_{0.27}Ti_{0.05})O_3$ antiferroelectric thick films prepared by tape-casing method [J]. Ceramics International, 2016, 42(11): 12537-12542.

[56] Shangguan D D, Duan Y N, Wang B L, et al. Enhanced energy-storage performances of (1-x)$PbZrO_3$-x$PbSnO_3$ antiferroelectric thin films under low electric fields [J]. Journal of Alloys and Compounds, 2021, 870: 159440.

[57] Li Y Q, Geng W P, Zhang L, et al. Flexible PLZT antiferroelectric film capacitor for energy storage in wide temperature range [J]. Journal of Alloys and Compounds, 2021, 868: 159129.

[58] Liu P, Fan B Y, Yang G, et al. High energy density at high temperature in PlZST antiferroelectric ceramics [J]. Journal of Materials Chemistry C, 2019, 7(15): 4587-4594.

[59] Zhao P F, Wang S B, Tang H, et al. Superior energy storage density and giant negative electrocaloric effects in $(Pb_{0.98}La_{0.02})(Zr, Sn)O_3$ antiferroelectric ceramics [J]. Scripta Materialia, 2021, 200: 113920.

[60] Gao P, Liu C, Liu Z H, et al. Softening of antiferroelectric order in a novel $PbZrO_3$-based solid solution for energy storage [J]. Journal of the European Ceramic Society, 2022, 42(4): 1370-1379.

[61] Tang Z H, Hu S C, Yao D J, et al. Enhanced energy-storage density and temperature stability of $Pb_{0.89}La_{0.06}Sr_{0.05}(Zr_{0.95}Ti_{0.05})O_3$ anti-ferroelectric thin film capacitor [J]. Journal of Materiomics, 2022, 8(1): 239-246.
